# Assessing the quality and standards of operative notes in general surgery; A teaching institute’s experience in Pakistan

**DOI:** 10.12669/pjms.40.8.9443

**Published:** 2024-09

**Authors:** Muhammad Danish Muneeb, Mirza Agha Naushad Baig, Muhammad Kamran, Shafaatullah Qudratullah, Muhammad Saddique Arain

**Affiliations:** 1 Muhammad Danish Muneeb, Baqai Medical University, Karachi, Pakistan; 2 Mirza Agha Naushad Baig, Baqai Medical University, Karachi, Pakistan; 3 Muhammad Kamran, Baqai Medical University, Karachi, Pakistan; 4 Shafaatullah Qudratullah, Baqai Medical University, Karachi, Pakistan; 5 Muhammad Saddique Arain, Baqai Medical University, Karachi, Pakistan

**Keywords:** General Surgery, Operative notes, Quality, Postoperative care, Resident surgeon, Duration of Surgery

## Abstract

**Objective:**

To evaluate the quality and standard of hand-written operative notes in a teaching institute.

**Methods:**

This prospective study was carried out in the department of surgery, Fatima Hospital, Baqai Medical University, from January 2023 till May 2023. One hundred fifty operative notes from general surgery domain were considered. These notes were evaluated according to the guidelines of Royal College of Surgeons, with added-on a few variables by the author.

**Results:**

All 150 notes were handwritten. Resident surgeon wrote the operative notes under the supervision of primary surgeon. There was a deficiency in mentioning medical record number, procedure starting time and duration of surgery. An important statement about the hemostasis is that it is secured-per-operatively was not documented. The residents were reluctant to explain the surgical procedures diagrammatically. The operative room number was missing in all notes. Post operative instructions lacked the information for nothing per oral, blood pressure, temperature, pulse rate, and input and output charting.

**Conclusion:**

It is observed that the operative surgical notes were however explainable about the procedure, but quality and standard was not matchable with that of Royal College of Surgeons notes. Hence, a lack of formal training for the resident surgeons in operative notes writing was observed. This study is a thought provoker to the surgeons and a guide to resident trainees, and hospital management to provide a handful operative notes writing theme in the form of performa provided in the department.

## INTRODUCTION

The description of the process of surgery and events related to the surgical procedure, are depicted, and documented in the form of operative notes. To audit, research and care of surgical patients and their relevant procedures, medical records availability is essential.^1^ Clinically, operative notes are vital for the patients’ safety and well-being.^2^,^3^ The increasing nature of population, diseases, hospitals, surgical practice is becoming more demanding and requiring more competition in lines of respect, fine and meticulous surgical technique with least margins of error. Therefore to deal with surgical error and complications, good practice requires proper documentation to cover medico-legal consequences in case.

There have been reported several deficiencies and weaknesses in documenting and writing operative notes particularly in surgical specialty.^4^ The tolerance on the other hand of the patients is another factor to be litigious. These operative notes should be true and error free. The General Medical Council guides and demands comprehensive documentation of surgical record by the surgeons, to achieve patients’ care through their track record and follow-up.^5^,^6^

Enhancing the quality and standards of operative notes directly relates to the standards of patient care and helps to reduce errors in surgery.^7^ Considering the protocols of writing operative notes set in Good Medical Practice, the Royal College of Surgeons of England (RCSEng)^8^ the operative notes performa was designed, including a couple of considerations by the author. Reluctant attitude in documenting and registering the operative notes, can let the cases open to litigation, in fact, the care and safety of patient can also be compromised.^9^

Therefore, to assess and determine the standards and quality of operative notes to be followed in our setup, we collected the data according to the format and guidelines recommended by the Royal College of Surgeons. The main purpose of this study was to assess the quality of operative notes and set a standard in our hospital setup.

## METHODS

A prospective analysis of the operative notes at Fatima Hospital, Baqai Medical University, was conducted from January 2023 till May 2023Guidelines from Royal College of Surgeons in filling the performa were considered. The data with percentages and frequencies were analyzed using SPSS software. The surgical unit of Fatima hospital, Baqai Medical University, comprises of departments namely General Surgery, Orthopedic Surgery, Pediatric Surgery, Plastic Surgery, Ear, Nose and Throat Surgery (ENT), and Urology.

### Ethical Approval

It was obtained from Ethical Review Board on 22^nd^ October 2021 with Reference number: BMU-FC/05-2021.

All the medium and major surgical procedures, performed in either spinal or general anesthesia were considered for the documentation. There are currently eight general surgeons while nine surgeons in other specialties listed above. The residents were asked to tick the variable options for documented or not documented. The variables include name, age, sex, date of the operation, time of the operation, the surgeon’s name, assistant’s name, scrub nurse’s name, theatre anesthetist’s name, mode of operation, use of antibiotic prophylaxis, name of procedure, incision (port site) used, operative findings, operative diagnosis, closure technique, estimated blood loss, secure of hemostasis, any extra procedure performed, postoperative care instructions, signature. Day cases and minor procedures done in local anesthesia were excluded from being documented in the performa. Major areas of weaknesses were highlighted, forwarded to the medical superintendent for application and further improvement. Some points were included in the performa including the operating room number for ethical purposes, diagrammatic representation of the cases for academic purposes.

### Inclusion criteria:

- Notes written by resident surgical trainees enrolled for master’s in surgery (MS) and Fellow of College of Physicians and Surgeons (FCPS) were included.

### Exclusion criteria:

- Notes written by house officers and by primary author were excluded from the study to remove any bias.

### Data analysis

Data was analyzed through SPSS (Statistical Package for Social Science) software version 21. Frequencies and percentages were considered.

## RESULTS

A total of 150 hand-written operative notes were reviewed from the department of general surgery Fatima Hospital, Baqai Medical University. All notes were written by the resident surgeons, under the supervision of the primary surgeon. Mostly the resident surgeons were from the second year of their residency program. The patient’s complete name is documented in all the operative notes initially, a token of their identification, however, the medical record number (MR no) listing lacked in 126 operative notes (Table-I). MR no. is one of the initial components for the identity and tracking and maintenance of a patient’s medical records.

**Table-I:** Operative Notes Data Not Documented.

S. No.	Data of Operative Notes	Number of Patients’ Operative Notes N=1500 (Not Documented)
	Medical record number	126 (84%)
	Duration of surgery	150 (100%)
	Operative room number	150 (100%)
	Secure of hemostasis	118 (79%)
	Diagramatic presentation	73 (49%)
	Preop antibiotic documentation	150 (100%)
	Complete postop notes	99 (66%)
	Signature of resident	87 (58%)

Duration of surgery was calculated by the time in and out of the patient, depicting that the surgeon worth the time of start and end of surgery. Duration of surgery is not documented in any of the cases (Table-I). The resident surgeon seems shy to document these variables. Mode of surgery, whether elective or emergency was mentioned (Table-II). Although operative findings are written in all cases, there is lack of diagrammatic representation of the operative procedure. The author assessed that in 73 of the cases diagrammatic representation was necessary and was not illustrated, however in 77 cases were not required. The name of the anesthetist is written in all operative notes, along with the mode of anesthesia and 78 (52%) of 150 notes the name of the technician who assisted in the case was present.

**Table-II:** Operative Notes Data Documented.

S. No.	Data of Operative Notes	Number Of Patient’s Operative Notes n=150 (Documented)
	Patient identification	150 (100%)
	Mode of surgery	150 (100%)
	Mode of anesthesia	150 (100%)
	Name of anesthetist	150 (100%)
	Identification of technician	78 (52%)
	Details of tissue removed	150 (100%)
	Details of blood loss	95 (63%)
	Closure technique	129 (86%)
	Documentation of mesh	150 (100%)

The operative room number was missing in all operative notes. Of 150 operations, none of the extra procedures were performed, apart from the primary and hence not documented. Details of the tissue removed, and fluid aspirated or drained were mentioned. Details of blood loss preoperatively were provided in 95 cases (63.3%), however, documenting in writing that hemostasis is secured after the completion of surgery and prior to closure, was surprisingly not mentioned in 118 cases (55.4%). The closure technique was defined properly with accompanying sutures in 129 notes (60%), but 21 (14%) of the notes relates to the deficiency in defining the name of the closure with suture. Provision of documentation for preoperative antibiotic cover was not provided in the written operative notes. DVT prophylaxis was not indicated in any of our surgeries, so not documented. Documentation of mesh in hernioplasties was documented in all the notes. Post-operative instructions were mentioned in all the notes; however, they were incomplete in 99 notes (66%), in defining the nothing per oral status, and documentation for temperature, pulse, respiratory rate and input and output charting Signature done by the resident trainee was lacking in 87 (58%) operative notes.

## DISCUSSION

In the training institute, the operative notes were usually written by resident surgeon, which was supervised by the primary surgeon, but it has been observed that the residents were not properly trained in writing the notes.^10^ This was commendable and there should be a structured guideline for writing operative notes.^11^ The documentation of variables provided in our performa were as low as compared to other similar studies.^12^ Even studies also defined a low quality and incomplete documentation of operative notes in the surgical practice.^13^ There was an error in remembering and documentation of name of technician assisted the case while other studies provide a better documentation in these aspects.^1^

The documentation record of the surgical team operated and assisted during the surgery is an asset of any procedure, guiding further the fate and management of surgery.^14^ The operative time must be documented Hence the duration of surgery was not noted, which is a healthy tool for a surgeon to review his quality of surgery. Duration of surgery time is nowadays a requirement for administrative purposes in the hospital.^15^ Primary surgeon’s name was properly documented in all the written notes, comparable to the study from United Kingdom.^16^

Written commentary for closure techniques, with its description and the nature and size of the sutures used, were missing in some of the notes. These types of variations were also prominent in studies from other parts of the world as well.^17^ The resident surgeons were also shy to document the intra-operative complications, which came into notice by inquiring the primary operating surgeons. Details of blood loss were provided, but the sentence of hemostasis secured at the end of the surgery was jeopardized, as it covers the surgeon as well as the surgery. This also depicts the pattern of complication during surgery which is worths defining.^18^ Nigerian research also classified the non-availability of per-operative complications documentations.^12^

Post-operative orders were followed according to the advice of primary surgeon, however, the instructions for nothing per oral, pulse, temperature, respiratory rate and input and output charting were missing. The type and amount of the fluid delivered to the patient was documented. Among the added variables, there was no documentary evidence on which operating room or table, the surgery was performed. The diagrammatic representation was least observed by the trainees’ surgeons. The resident surgeons’ signatures were also missing in a few operative notes. The medical record number (MR no), which was scarcely defined in our operative notes, has always been essential in identifying and tracking patients’ records. Studies also show that the absence of patient identity might place the patient in jeopardy to be misplaced.^19^ Therefore, the role of providing a structured performa, specifying the components which were usually less considered by the trainees, can be highlighted, hence can improve the quality of operative notes.^20^

Several studies, which compared their performa with the standard one, checked the compliance for the instructions or variables present in their operative notes writing, and proved usually to be comparably well.^21^,^22^ Proceeding further than this concept, we also compared the quality of operative notes by further adding variables, and also by quality of postoperative orders provided. This can enhance the quality of operative notes instructions.

Incomplete handwritten operative notes can weaken a surgeon’s security as well.^18^ Electronic system availability has provided a great benefit in documentation and hence the compliance of the standard of documentation^23^,^24^ and training of resident trainees^25^ Still seeking forward for electronic documentation, we usually comply with the hand-written surgical notes, hence their further improvement.

## CONCLUSION

To conclude, it is worth mentioning that a standard surgical procedure is reflected by the good quality and standard of operative notes. We suggest that a written operative notes performa with defined variables should be provided through proper hospital administration to the surgery department, so that they work as memory aid for the surgeons and trainees and help in proper training of the resident surgeons.

### Author’s Contribution:

**MDM, MANB:** Idea, design, statistical analysis.

**MK, MDM:** Data collection and manuscript writing and editing.

**MDM, SQ:** Drafting and revision of article.

**MSA, MDM, MANB:** Critical analysis and final approval.
